# Mobile Health Solutions for Prostate Cancer Diagnostics—A Systematic Review

**DOI:** 10.3390/clinpract13040078

**Published:** 2023-07-28

**Authors:** Masood Moghul, Walter Cazzaniga, Fionnuala Croft, Netty Kinsella, Declan Cahill, Nicholas David James

**Affiliations:** 1 Department of Urology, The Royal Marsden NHS Foundation Trust, London SW3 6JJ, UK; 2 Division of Radiotherapy and Imaging, The Institute of Cancer Research, London SW3 6JB, UK

**Keywords:** prostate cancer, mobile clinic, community setting

## Abstract

Prostate cancer, the most common cause of cancer in men in the UK and one of the most common around the world to date, has no consensus on screening. Multiple large-scale trials from around the world have produced conflicting outcomes in cancer-specific and overall mortality. A main part of the issue is the PSA test, which has a high degree of variability, making it challenging to set PSA thresholds, as well as limited specificity. Prostate cancer has a predisposition in men from black backgrounds, and outcomes are worse in men of lower socioeconomic groups. Mobile targeted case finding, focusing on high-risk groups, may be a solution to help those that most need it. The aim of this systematic review was to review the evidence for mobile testing for prostate cancer. A review of all mobile screening studies for prostate cancer was performed in accordance with the Cochrane guidelines and the PRISMA statement. Of the 629 unique studies screened, 6 were found to be eligible for the review. The studies dated from 1973 to 2017 and came from four different continents, with around 30,275 men being screened for prostate cancer. Detection rates varied from 0.6% in the earliest study to 8.2% in the latest study. The challenge of early diagnosis of potentially lethal prostate cancer remains an issue for developed and low- and middle-income countries alike. Although further studies are needed, mobile screening of a targeted population with streamlined investigation and referral pathways combined with raising awareness in those communities may help make the case for screening for prostate cancer.

## 1. Introduction

Prostate cancer is the most common cancer in men in the UK, with over 50,000 new cases diagnosed and 12,000 lives lost to prostate cancer in the UK each year [1]. This is of particular concern for black African and African Caribbean men, who carry a one in four risk of developing prostate cancer (compared with one in six men overall) [2], and for men with a family history of the disease. As cancers tend to arise in the peripheries of the prostate gland, there may be few or no symptoms, even from locally advanced disease, until very late on. Even with symptoms, they can easily be conflated with benign disease or ignored as a typical feature of the ageing process.

Early diagnosis and treatment are potentially lifesaving for a range of conditions, with late presentation being a major cause of death from cancer. A range of barriers to accessing healthcare have been identified, including low education levels [3] (which may result in a lack of knowledge of relevant symptoms), logistical difficulties in accessing healthcare (for example, due to anti-social working hours and geographical locations) [4], and financial considerations (particularly in insurance-dependent healthcare systems). These issues are often exacerbated in low- and middle-income countries (LMICs), as demonstrated by the severe burden of prostate cancer in sub-Saharan Africa [5,6], which is thought to be increasing [5]. The wider effects of health inequalities affect all in society, with a recent Deloitte report estimating the annual healthcare US expenditure due to health inequities at $320 billion, which is set to rise to $1 trillion or more by 2040 [7].

Despite many advances in the field of early diagnosis of prostate cancer, significant issues remain. PSA testing remains at the forefront of early diagnostic testing despite its unreliability as a screening tool for prostate cancer, making its widespread use contentious. A key issue in using PSA as a screening tool comes from the definition of what a ‘normal’ PSA is. Oesterling’s age-specific PSA ranges from 1993 are still in use today [8]. PSA is not only elevated in prostate cancer but can also be elevated due to benign growth of the prostate, urinary tract obstruction, infection, or prostatic inflammation. Having a higher PSA threshold for investigation increases the positive predictive value for detecting prostate cancer but lowers the negative predictive value, and vice versa. For example, a study by Gerstenbluth documented a PPV of 98.5% for PSA in detecting prostate cancer with a PSA of 50 ng/mL or above [9], but a threshold this high, while largely eliminating false positives, would also miss the majority of clinically significant cancers.

The evidence for screening remains controversial. The Prostate, Lung, Colorectal, and Ovarian (PLCO) Cancer Screening Trial [10] from the USA looked at systematic vs. opportunistic screening in over 76,000 men. Using a PSA threshold of over 4 and 7–10 years of follow-up, no significant difference in mortality from prostate cancer was found between the screened and non-screened arms. However, the study had high rates of contamination between the two groups, with an estimated 74% of the control group subject to at least one routine PSA test during the trial (95% in the intervention arm) and around 50% of men in the control group tested each year [11].

The European Randomised Study of Screening for Prostate Cancer (ERSPC) has been studying 186,000 men aged 55 to 69 in Europe since 1992. In its 16th year of follow-up [12], results show a 20% reduction in cancer-specific survival, and the number needed to treat is now similar to that observed in breast cancer screening [13], strengthening the argument for prostate cancer screening. A total of seven studies in different regions of Europe were included, with each study using different screening criteria, treatment protocols, interval timing, and PSA thresholds (between 3 ug/L and 4 ug/L), which threatens the credibility of its conclusions.

The use of pre-biopsy MRI scanning has enhanced diagnostic rates and reduced unnecessary biopsies [14,15], and the transition from transrectal to transperineal approaches has further reduced morbidity from biopsies [16]. While these achievements are notable, case finding remains an issue, with nearly 20% of men in the UK diagnosed with metastatic disease [17]. As these men account for at least half of those who eventually die from prostate cancer, reducing the rate of metastatic presentation is key to reducing mortality. New biomarkers once heralded as potential game changers have not yet successfully displaced the use of PSA in routine testing. Liquid biopsies may yet yield further benefit but remain some way from routine clinical use.

The National Institute for Health and Care Excellence (NICE)’s guidelines in the UK suggest a risk-stratified approach along with patient counselling. The guidelines recommend consideration of a PSA test for men with lower urinary tract symptoms, haematuria, or erectile dysfunction [18]. As already noted, there is a poor correlation between symptoms and the presence of prostate cancer [19], with many men diagnosed with clinically significant prostate cancer experiencing no symptoms. Routine systematic screening is also not currently recommended by either the AUA [20] or EAU [21].

Although findings from large-scale prostate cancer screening trials have found equivocal results, targeted screening programmes for high-risk populations may yet yield benefits. Mobile health units may enable increased access for groups that otherwise struggle to access healthcare. This may be especially advantageous in LMICs, where healthcare infrastructure is even more sparse. Screening for a range of diseases in combination with health promotion may increase economic viability and provide a wider range of health benefits for these populations. Mobile screening for other diseases (e.g., HIV) has already shown feasibility and economic viability [22], and combining mobile health solutions with a risk-stratified approach targeting high-risk populations may help to crack the nut that is early diagnosis of prostate cancer.

The aim of this systematic review was to review the evidence for mobile testing for prostate cancer.

## 2. Methods

The review was performed in accordance with the Cochrane guidelines and the Preferred Reporting Items for Systematic Reviews and Meta-Analyses (PRISMA) statement [23].

The following bibliographic databases were searched for studies on mobile screening for prostate cancer up to August 2022: MEDLINE (PubMed), EMBASE, Cochrane Central Register of Controlled Trials (CENTRAL), Web of Science, and the WHO Global Health Library. The search was conducted on 1 August 2022.

No restrictions were placed on language, study design, study location, or participants. All studies that offered tests for prostate cancer using mobile methods were included. Published articles in peer-reviewed journals or thesis dissertations were included, as well as hand-searching individual urological journals, citations, and reference lists. Conference abstracts/posters, review articles, and opinion pieces were excluded. Search terms included (but were not limited to) the following: ‘prostate’, ‘cancer’, ‘screening’, ‘mobile’, ‘movable’, and ‘traveling’. Boolean operators (AND, OR) were employed to augment the search process. Details of the search strategy are included in Supplementary File S1.

Titles and abstracts were reviewed to assess potential studies, with full texts of eligible articles screened by one author and verified by a second. In the event of any disagreements between the authors, they were resolved by discussion or consultation with a third author. Two authors independently reviewed all full-text articles, with data extracted onto a standardised spreadsheet and validation by each reviewer of the other’s data. 

Data extracted from the studies included the following: study design, country, population details, number of men screened, type of mobile screening facility (e.g., mobile van or other type of temporary facility), and performance measures, including the rate of screening tests, rate of follow-up loss (i.e., patients referred for further investigations but lost to follow-up), number of detected cases, and characteristics of detected cases, including staging and extent of disease. The results were synthesised into a tabulated format.

A statistical analysis or meta-analysis was not possible due to the heterogeneity of study designs and endpoints. Studies were, therefore, analysed qualitatively in terms of various key parameters, such as study type, design, length, men screened for prostate cancer, average age, method of mobile testing, prostate cancer test, PSA thresholds, percentage investigated, further investigations performed, loss of follow-up, biopsy rates, prostate cancer detection rates, stage of disease, and grade of disease. The quality of the studies was assessed using the MMAT (Mixed Methods Appraisal Tool) [24], with independent verification by two authors.

## 3. Results

**Figure 1:**
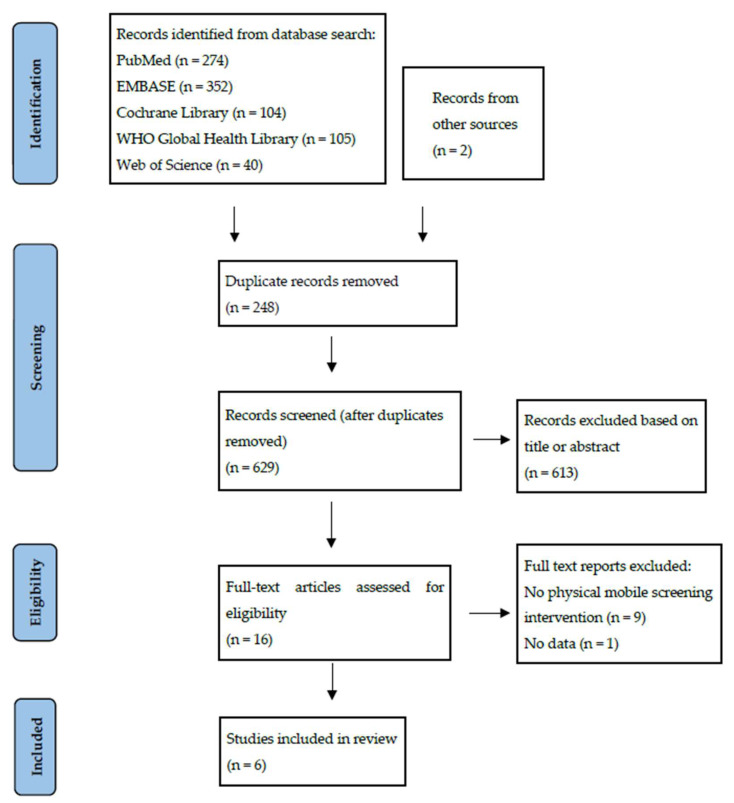
A PRISMA flow diagram of studies for inclusion in a review of mobile screening for prostate cancer.

The initial search identified 875 articles, with 2 additional articles identified through other means. After excluding duplicates, 629 unique articles were identified. Analysis of titles and abstracts narrowed this down to 16 articles, for which the full text was reviewed. Of these, six studies met the inclusion criteria. A PRISMA flow diagram illustrating the search process is shown in Figure 1.

A summary of these six studies (published between 1973 and 2020) is shown in Table 1. Overall, at least 30,275 men were screened (with Lynch et al. [25] not reporting individual numbers of men and women screened). Three studies were purely focused on screening for prostate cancer, with the other three being multi-cancer screening studies. Three were based in the USA (of which one targeted the African American community [26]), with one each from Africa, the Far East, and South America. The study designs are reported in Table 1, but the majority were cross-sectional, with results published of a descriptive nature. One study had a prolonged follow-up with prospective data. Performance measures are also shown in Table 1; however, the initial studies were more focused on proving the proof of concept and the calculation of potential health economics. Due to the prolonged period between studies, the type of screening varied drastically, beginning with just a rectal exam before moving onto transrectal ultrasound and then finally PSA blood tests combined with a digital rectal exam. The overall score from the methodological quality review was 76% across the six studies assessed, indicating a reasonable level of quality, although direct comparisons are challenging due to heterogeneity.

The interaction between fixed healthcare assets (hospitals, clinics, etc.) and the mobile service varied between the studies depending on existing infrastructure and the type of community being targeted (rural versus urban). Ashorobi et al. [26] began the project in static centres before moving onto a mobile service, while Jatho et al. [27] ran simultaneous hospital and mobile-based screening services, with comparisons between the two showing a significantly higher detection rate of prostate cancer in the mobile screening setting (8.2% vs. 7.1%, chi-squared test: *p* < 0.05).

Health economic data from the included studies are limited. However, Jatho et al. [27] noted that with an average screening cost of $30 USD, treatment costs for treating advanced disease for one patient are 600× higher ($18,000 USD).

## 4. Discussion

Major screening trials for prostate cancer have been carried out in unselected populations in high-income countries with generally good healthcare provision, often with men from the upper end of economic groups overrepresented and with disproportionately lower levels of ethnic minorities [10,28]. Worse outcomes for prostate cancer in ethnic minorities and lower socio-economic groups highlight the importance of investigating and developing methods to overcome these barriers [29,30,31]. In LMICs, there are different issues, with poorer access to healthcare and lower education levels leading to late-stage presentations being the norm. 

Even in high-income settings, too many patients are still found to have cancer after an emergency presentation—around 30% for cancers overall in the UK [32] (although the figure for prostate cancer is closer to 10% [33,34]). For men diagnosed with prostate cancer, 8% were found to have attended emergency departments at least once in the 30 days prior to diagnosis [35]. Furthermore, presentation at a late stage means that treatment is less likely to be successful and will certainly carry more morbidity for the patient and cost for the healthcare system. 

The NHS sees many patients accessing cancer care at a late stage. Reducing this trend is a key objective of the NHS Long Term Plan [36], with similar goals within the US Cancer Moonshot programme [37] and the European Union’s Cancer Beating Plan [38]. The recent COVID-19 pandemic has led to a sharp decline in referrals for cancer diagnostics [39], suggesting that there may be a growing reservoir of undiagnosed cancer in the community. Mobile clinics can potentially be a model for alleviating this, where their presence in a community setting makes ‘seeking advice on health issues’ less daunting, more normal, and easily accessible.

A key part of any mobile screening service is raising awareness among the target community. This is particularly important as these initiatives are often new and patients may not be fully aware of the services offered. In the earliest studies, Lynch et al. [25] mentioned using local community leaders and physicians to coordinate this. Faria et al. [40] in Brazil had a programme for raising awareness that was conducted two weeks prior to the mobile service coming to a particular area. This consisted of the distribution of printed materials, messaging via local radio stations, and making children in schools more aware (presumably to then inform parents). Ashorobi et al. [26] used multiple media outlets as well as community engagement via churches, community centres, and barber shops, with a specific focus on targeting men from the African American community. Jatho et al. [27] in Uganda utilised a similar mixture of media messaging with local community infrastructure. 

Studies prior to PSA development clearly suffer from much lower detection rates. Once PSA testing is involved, the detection rates begin to approach those of other screening studies; for comparison, the PLCO study had a detection rate of around 1.4% after one screening round [10], and the first screening phase of the CAP trial had a rate of 3.3% [41] (18-month follow-up).

Despite the consistency in the utilisation of PSA in the three later studies, the investigation criteria still differ widely due to PSA thresholds and the use of free-to-total ratios in one study and no mention of PSA thresholds in another. All studies used digital rectal examinations of the prostate as a screening tool (with the Japanese study using a transrectal ultrasound scan), still showing their potential importance, particularly in LMICs. Faria et al. [40] showcased this further, with 19.7% of their prostate cancer diagnoses coming from men with an abnormal DRE but PSA levels below threshold. 

Notably, Jatho et al. [27] compared diagnostic rates from their mobile service against hospital-based screening and found them to be significantly higher, suggesting that the mobile-targeted approach can provide improved access for higher-risk men. The 8.2% detection rate from this study was strikingly higher than the other studies, from which rates were calculable, perhaps due to the increased risk seen in black men. Although there are vast ethnic variations within Africa that as yet remain incompletely understood, targeted screening of high-risk populations through a mobile approach may increase detection rates in larger-scale studies.

Contamination is a concern in all screening studies. With the limited data available, prior PSA testing was only documented in the Brazilian study, with around 30% of men having had a previous DRE and 30% having had a previous PSA. Furthermore, some studies did not separate results from fixed and mobile clinics, making interpretation challenging. Loss of follow-up is also an issue, particularly in the study by Ashrobi et al. [26], with a 60% loss of patients after a referral was recommended, which may have adversely affected their detection rate.

A key principle of mobile screening has been a multimodal publicity campaign prior to the arrival of the mobile screening service. Of note, the use of radio broadcasts and schoolchildren in LMICs may be important techniques to circumvent the issue of low literacy rates. Other awareness-raising methods (flyers, telemedicine, social media, etc.) may also be important in specific communities. The multipronged approach of increasing awareness in the desired community, combined with providing ease of access to the screening service, seems to be a successful approach for maximising engagement, although there is limited information about the demand generated from these studies.

Although mobile services have been shown to have many uses, they may not be appropriate for all areas. Large buses or trailers may have more logistical challenges in urban areas than rural ones. Combining approaches with fixed clinical assets (e.g., clinic rooms or nursing rooms) with mobile services is likely to be beneficial when covering wide areas with diverse infrastructure set-ups. A further possibility is the creation of ‘pop-up‘ clinics in units such as unused shops or community centres. Mobile screening operations should strive to minimise potential delays in the referral pathway and the loss of follow-up that may occur due to delays between the screening test and the dissemination of results and the availability of further tests and treatments. The use of point-of-care blood testing to provide rapid PSA results can improve diagnostic times further and reduce the administrative burden resulting from delayed laboratory results. A fully integrated plan with aspects of education, awareness-raising, rapid diagnosis, and treatment is required. 

Developments in pre-biopsy multiparametric MRI scans and evolving biopsy techniques (transperineal with MRI targeting) are likely to increase the detection rates of clinically significant cancers further, although applicability in LMICs may be limited at present. These developments may increase upfront costs (e.g., MRI scans) but reduce the rates of unnecessary biopsies and clinically insignificant disease [14,15]. Further studies looking at mobile techniques in conjunction with modern rapid diagnostic practises (including novel biomarkers and genetics) may yield further information and make a more comprehensive economic argument for targeted screening for prostate cancer. Limitations of these studies include a lack of information about the demand generated from awareness campaigns, cancer staging and grading information, and patient satisfaction data to showcase patient perceptions of mobile screening.

**Table 1:** Illustrating key findings from all studies included in the review.

Author	Year	Country	Study Type & Quality Score	Design	Length	Men Screened for PCa	Av. Age	Method of Mobile Testing	Prostate Cancer Test	PSA Thresholds	% Further Investigated	% Loss of Follow-Up	Biopsy Rates	% PCa Detection Rates (n)	Stage of Disease	Grade
Lynch et al. [25]	1973	USA	Quantitative Descriptive MMAT: 40%	Multi-cancer	1 year	3040 men and women	60	Custom built trailer	Not specified, likely rectal exam	n/a	n/a	n/a	n/a	2 (rate not calculatable)	n/a	n/a
Lynch et al. [42]	1976	USA	Quantitative Descriptive MMAT: 60%	Multi-cancer	4 years	1984	n/a	Custom built trailer	Not specified, likely rectal exam	n/a	n/a	n/a	n/a	1 (0.05%)	n/a	n/a
Watanabe et al. [43]	1984	Japan	Cross-sectional MMAT: 60%	PCa only	6 years	325 men in trials, then 1071 on mobile unit (total 1396)	n/a	Preliminary model, then customised bus	Transrectal ultrasound	n/a	n/a	n/a		0.6% (8)	n/a	n/a
Faria et al. [40]	2010	Brazil	Cross-sectional MMAT: 80%	PCa only	3 years	17,571	61.2	Mobile unit	PSA (free and total), DRE	initially >4, then >2.5 ng/mL if %fPSA ≤ 15%	16.1% (2841)	19.4%	9.4% (1647)	3.1% (552) on first screen, overall rate including men screened multiple times: 3.7% (652)	76.4% T1 17% T2	67.5% GG1 32.5% ≥ GG2
Ashorobi et al. [26]	2017	USA	Mixed: cross sectional and prospective MMAT: 80%	PCa only	6 years	4420	52	Initially using fixed locations then bus.	PSA, DRE	>2.5 ng/mL	14% (609)	60%	2.6% (116)	3.3% (144) overall	n/a	n/a
Jatho et al. [27]	2020	Uganda	Cross-sectional MMAT: 60%	Multi-cancer	3 years	4904	n/a	Mobile van and community-based assets.	PSA, DRE	n/a	n/a	n/a	n/a	8.2% (404)	n/a	n/a

## 5. Conclusions

Despite increasing research aimed at finding novel methods of targeting patients for disease screening, there is limited information on the use and efficacy of mobile screening for prostate cancer. Although intuitively mobile screening combined with an awareness-raising drive would seem to be a useful approach, the complexities of cancer screening in different communities, different countries, and differing types of residential areas make drawing blanket generalisations difficult. Despite this, some conclusions can be drawn. The feasibility of mobile screening for prostate cancer shows it is widely applicable across different countries, in both developed and LMICs. Due to the main cost of such services being staff, they may even be more economical in LMICs, where staffing costs are lower, although there may be increased challenges to further investigation and treatments.

## Data Availability

No new data were created or analyzed in this study. Data sharing is not applicable to this article.

## Supplementary Materials

The following supporting information can be downloaded at: https://www.mdpi.com/article/10.3390/clinpract13040078/s1, Supplementary File S1: PRISMA Checklist.
